# Breast Tumor Tissue Image Classification Using Single-Task Meta Learning with Auxiliary Network

**DOI:** 10.3390/cancers16071362

**Published:** 2024-03-30

**Authors:** Jiann-Shu Lee, Wen-Kai Wu

**Affiliations:** Department of Computer Science and Information Engineering, National University of Tainan, Tainan 700, Taiwan; m10859015@stumail.nutn.edu.tw

**Keywords:** single-task meta learning, auxiliary network, image classification, medical images

## Abstract

**Simple Summary:**

Breast cancer is one of the deadliest forms of cancer, but early and accurate diagnosis can significantly boost patient survival rates. Traditional classification models struggle with the diverse characteristics of breast tumor pathology images, leading to misdiagnoses. To tackle this challenge, our study introduces a new model, combining Single-Task Meta Learning and an auxiliary network to enhance diagnosis accuracy. This innovative approach enables the model to better generalize, recognizing and categorizing varied image data effectively. Our findings reveal that it surpasses current methods, boosting classification accuracy in complex tasks by at least 1.85%. Moreover, a 31.85% increase in the Silhouette Score for the model’s learned features indicates an improved ability to identify critical differences between tumor types. This advancement not only promises more accurate early diagnoses but also holds the potential to save lives, showcasing a significant leap forward in the clinical management of breast cancer.

**Abstract:**

Breast cancer has a high mortality rate among cancers. If the type of breast tumor can be correctly diagnosed at an early stage, the survival rate of the patients will be greatly improved. Considering the actual clinical needs, the classification model of breast pathology images needs to have the ability to make a correct classification, even in facing image data with different characteristics. The existing convolutional neural network (CNN)-based models for the classification of breast tumor pathology images lack the requisite generalization capability to maintain high accuracy when confronted with pathology images of varied characteristics. Consequently, this study introduces a new classification model, STMLAN (Single-Task Meta Learning with Auxiliary Network), which integrates Meta Learning and an auxiliary network. Single-Task Meta Learning was proposed to endow the model with generalization ability, and the auxiliary network was used to enhance the feature characteristics of breast pathology images. The experimental results demonstrate that the STMLAN model proposed in this study improves accuracy by at least 1.85% in challenging multi-classification tasks compared to the existing methods. Furthermore, the Silhouette Score corresponding to the features learned by the model has increased by 31.85%, reflecting that the proposed model can learn more discriminative features, and the generalization ability of the overall model is also improved.

## 1. Introduction

According to the World Health Organization’s cancer statistics from 2018 [1], there were approximately 18 million cancer cases, among which breast cancer accounted for about 2 million cases, representing a significantly high proportion. Moreover, compared to other cancers, breast cancer also has a higher mortality rate, constituting about 6.5% of all cancer-related deaths. Therefore, research related to breast cancer is given considerable attention in the medical field. Accurate diagnosis has always been one of the critical issues in cancer research. The diagnostic methods for breast cancer include the following: cancer biomarker screening, X-ray imaging, ultrasound imaging, magnetic resonance imaging (MRI), thermal imaging, and histopathological slide examination. Despite the availability of various diagnostic methods for breast cancer, only histopathological slide examination can confirm whether a patient has cancer. Currently, the examination of histopathological slides largely relies on pathologists to delineate tumor regions and perform counting, which is time-consuming and challenging to achieve comprehensive statistics for a slide image that could be as large as 2 GB. However, determining the presence of breast cancer and the cancer grading from histopathological slides requires experienced histopathologists. If the pathologist is in a poor mental state, it could lead to incorrect judgment, potentially causing patients to miss the golden window for treatment. Therefore, there is an urgent need in current clinical pathology diagnosis for the development of automated assistance analysis and diagnostic tools for histopathological slide images.

A computer-aided diagnosis (CAD) system [2,3] involves leveraging computer-generated outputs as support tools for clinicians to make medical diagnoses. The urgently needed functionality in current CAD systems for histopathological slide images is the binary classification of pathology images into benign and malignant categories, followed by subclassification within these categories. According to the information described in [4], benign tumors are classified into fibroadenomas (Fs), tubular adenomas (TAs), adenosis (A), and phyllodes tumors (PTs); malignant tumors are classified into lobular carcinoma (LC), papillary carcinoma (PC), mucinous carcinoma (MC), and ductal carcinoma (DC). Traditional CAD systems primarily use image processing techniques to extract features from pathology images [4,5,6,7,8,9,10], such as Histograms of Oriented Gradients (HOGs), Local Binary Patterns (LBPs), Haar Discrete Wavelet Transform (HDWT), Completed Local Binary Patterns (CLBPs), Local Phase Quantization (LPQ), and Fractal Dimension. Although these techniques can extract features for classification, their performance is limited due to the manually designed feature extraction methods. This limitation has been overcome by the use of Convolutional Neural Networks (CNNs) in deep learning. Over the past decade, CNNs have garnered significant success in the realms of image and video analysis, drawing the attention of researchers focusing on pathology image analysis.

Motlagh et al. [11] introduced the use of Inception and ResNet architectures for distinguishing cancerous microscopic images, establishing an automated system for detecting breast tumors and classifying their subtypes. However, the reliance on pre-existing CNN models for feature extraction and classification in the aforementioned methods does not fully accommodate the unique attributes of pathological images, leading to inherent limitations in classification efficacy. To address this, Jiang et al. [12] proposed a novel CNN framework specifically tailored for the classification of breast cancer histopathology images by incorporating the compact SE-ResNet module, dubbed the Breast Cancer Histopathology Image Classification Network (BHCNet). This innovative model facilitates the automated categorization of breast cancer histology images into benign, malignant, and eight distinct subtypes.

In the task of classifying breast tumor pathology images, due to the inherent variability in the characteristics of each pathology image, such as staining differences, magnification levels, instrument characteristics, slice positioning, and individual tissue variations [13,14,15], it often occurs that a pathology tissue classification model trained by a unit may only perform well on data similar to the training samples. When faced with data whose image characteristics significantly differ from the training samples, the model’s performance may not meet expectations. This poses a considerable challenge for the clinical application of breast tumor pathology classification CAD systems. Therefore, there is an urgent need in clinical applications for a pathology tissue classification model with excellent generalization ability.

Although the aforementioned BHCNet [12] achieves commendable classification performance, there remains room for improvement in its generalization capabilities. A viable approach to enhancing the generalization ability of breast pathology image classification models involves augmenting the classification network with auxiliary networks to improve its classification performance on previously unseen data, thereby boosting the network model’s generalization capability in classifying breast tumor pathology images. Following this approach, to maximize the overall model’s generalization ability as much as possible, three key components must be emphasized: the main network model, the auxiliary network model, and the learning strategy for the auxiliary network model. Based on this perspective, this study introduces a new breast pathology image classification model named STMLAN (Single-Task Meta Learning Auxiliary Network). This model employs ResNeXt [16] as the main network model, which is a classification model pre-trained on the ImageNet dataset [17] and demonstrates strong classification capabilities. STMLAN utilizes MetaOptNet [18], known for its analytical optimization capabilities, as the auxiliary network. Furthermore, to further optimize the feature space, this study proposed a novel learning strategy, Single-Task Meta Learning, serving as the learning approach for the auxiliary network, thereby further enhancing the overall classification network model’s generalization ability. The outcomes of our experiments demonstrate that our approach surpasses other CNN-based classification techniques in accurately identifying benign versus malignant tumors and in the categorization of eight distinct subcategories. Furthermore, through ablation studies, it has been confirmed that using ResNeXt as the main network model yields the best performance, while MetaOptNet demonstrates the most superior performance along with the auxiliary network. Additionally, methods such as feature data visualization and Silhouette Scores corroborate that employing the Single-Task Meta Learning strategy to train the auxiliary network significantly enhances the main network’s feature embedding model. This training enables the extraction of more discriminative and broadly applicable high-level features from breast tumor pathology images, affirming the efficacy of our method.

The rest of the paper is organized as follows: Section 2 provides a retrospective introduction to auxiliary networks and meta learning. Section 3 is dedicated to the proposed STMLAN and its training. In Section 4 and Section 5, the dataset and experimental results comparisons are given and concluded.

## 2. Related Work

### 2.1. Auxiliary Network

Both human learning and artificial learning require data or examples to generate learning outcomes, but human learning also involves deductive reasoning, which encompasses a wide range of cues, i.e., relevant information fragments, to enhance human understanding [19,20]. Educational research has found that the use of interactive hints is a useful tool for improving students’ learning efficiency [21], and research on learning from hints has been discussed and incorporated into the training of neural networks [22]. Suddarth and Kergosien [23] proposed a “rule-injection hints” method, which adds additional supervisory neurons to the network, achieving shorter training times and enhanced model generalization performance. Besides the existing approach of adding neurons, hints can also be introduced in the form of auxiliary networks. Auxiliary networks can be further differentiated based on their operational modes into data-auxiliary and loss-auxiliary. 

Pan et al. [24] were the first to propose the idea of data-auxiliary, with subsequent scholars presenting various specific implementation methods. Rusu et al. [25] introduced PNNs (Progressive Neural Networks), starting with training a network model for the first task. After training, the weights are fixed, and then the second task is trained, where the input for the second task includes the output of layers trained previously. The merging of layers employs dimensionality reduction techniques. Following the same rule, by utilizing the features from previous networks to assist with the prediction of the last network, it allows the last network to learn information from other auxiliary networks, thereby offering an opportunity for the further enhancement of performance. Tzeng et al. [26] incorporated MMD (Maximum Mean Discrepancy) as a distance metric to aid network learning. Assuming there are two datasets, A and B, where dataset A contains labeled data and dataset B contains unlabeled data, the process first extracts features through a neural network. Then, different network layers are used to obtain the features of A and B, respectively. Finally, MMD is used to calculate the feature distance between A and B as the loss function. The aim is to utilize dataset A to assist the unlabeled dataset B, thereby enhancing the performance of dataset B. Long et al. [27] introduced MK-MMD, which modifies the original approach [26] of computing MMD using only the last layer to now utilize the last three layers. Ganin et al. [28] proposed a novel model, DANN (Domain-Adversarial Neural Network), with the goal of using labeled dataset A to assist in classifying the unlabeled dataset B. What sets it apart is the use of two classifiers: the Label Predictor and the Domain Classifier. The Domain Classifier is designed to classify whether input features belong to dataset A or B. To confuse the network and make it unable to distinguish whether data features come from dataset A or B, thereby aligning the feature distribution of dataset B with that of dataset A, the gradients of the Domain Classifier pass through a gradient reversal layer during backpropagation, enhancing the network’s classification performance.

Data-auxiliary involves using an additional dataset to assist the learning of the original dataset, while loss-auxiliary pertains to assistance with the loss function, meaning that training uses only a single dataset but employs two loss functions to guide model learning. Bazi et al. [29] utilized an auxiliary network comprising a layer of 3 × 3 convolution, GAP (Global Average Pooling), and Dropout, and to prevent gradient vanishing during transfer learning, they employed root-mean-square propagation (RMSprop) as the optimizer. Jin et al. [30] suggested the use of an additional auxiliary network at the final layer for object detection, where this auxiliary network does not perform residual connections or acquire anchor boxes from previous layers, employing this method to enhance the features of the last layer and improve performance. Yu et al. [31] divided the fully connected layer into two parts: the main classification and auxiliary classification. The main classification part is responsible for instrument classification, while the auxiliary classification focuses on grouping instruments, using the grouping of instruments to provide the network with more information, thereby enhancing classification performance. As this study does not use an additional dataset to assist the learning of the original dataset but rather aims to optimize the classification performance of the original classification network, the auxiliary network used belongs to the category of the loss-auxiliary network.

### 2.2. Meta Learning

Deep learning involves learning from vast amounts of data through deep neural networks, focusing on extracting features and making predictions based on those features. Meta Learning is about learning how to learn. It aims to design models that improve their learning capability with experience, adapting to new tasks efficiently with minimal data. This issue has garnered considerable focus within the machine learning field, where few-shot learning is approached as a meta learning challenge (for instance, [32,33,34]). The primary aim is to optimize the generalization error across a spectrum of tasks that only provide a limited number of training samples. Commonly, these methodologies consist of an embedding model responsible for translating the input domain into a discernible feature space, accompanied by a base learner that interprets this feature space in terms of task-specific variables. The overarching goal of meta learning is to develop an embedding model that enables the base learner to achieve broad task generalization. Considering the inherent capability of meta learning to reduce generalization error, this study incorporates meta learning strategies into the training of the auxiliary network, thereby further enhancing the classification performance of the main network. Originally, meta learning was conducted with a focus on cross-task training. However, to align it with the requirements of this study, we adapted it to a training approach oriented towards cross-data characteristics, which we refer to as Single-Task Meta Learning.

## 3. Proposed Method

### 3.1. System Architecture

**Figure 1:**
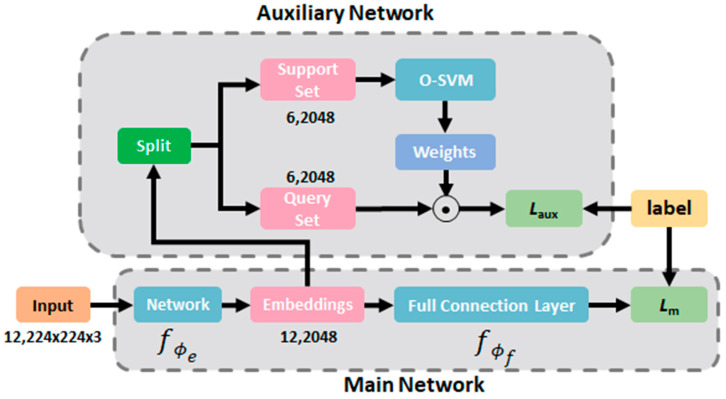
System architecture of the proposed STMLAN.

Figure 1 illustrates the architectural diagram of the STMLAN system proposed in this study. The architecture is primarily divided into two components: the main network (pre-trained) and the auxiliary network. The final layer features of the main network serve as the input for the auxiliary network, which aids in optimizing the feature space of the main network. The role of the auxiliary network is solely to assist in optimizing the main network during the training phase, while only the main network is utilized during the inference stage.

### 3.2. STMLAN

In recent years, several transfer learning architectures have emerged, including SE-ResNet [35], ResNeXt [16], and DenseNet [36], with ResNeXt demonstrating superior performance among them. ResNeXt is designed around a central principle of replicating a building block that aggregates a collection of transformations sharing the same topology. This approach yields a uniform, multi-branch structure characterized by minimal hyper-parameter configuration. This design introduces “cardinality” (the number of transformation sets) as a crucial dimension alongside depth and width. Empirical results on the ImageNet-1K dataset indicate that, even when complexity is constrained, an increase in cardinality enhances classification accuracy. Furthermore, boosting cardinality proves to be a more efficient way to augment model capacity than increasing depth or width. Consequently, this study employed a pre-trained ResNeXt model as the main network.

**Figure 2:**
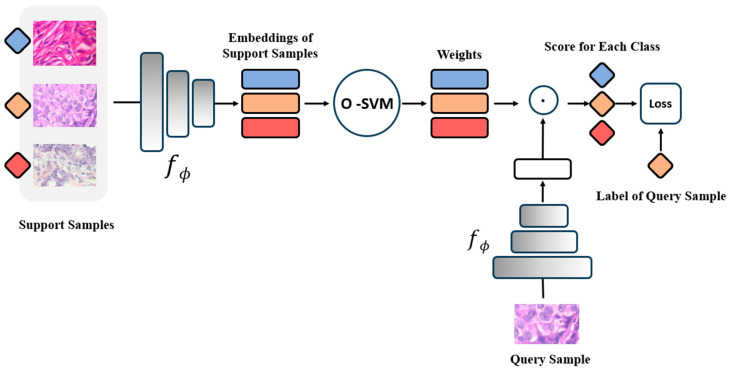
The structure of MetaOptNet using a 1-shot 3-way classification task for example.

Kwonjoon et al. introduces the MetaOptNet model [18] (as shown in Figure 2), leveraging the convex optimization neural network, OptNet [37], developed by Brandon et al. based on Quadratic Programming, to realize a differentiable linear SVM classifier denoted as O-SVM. This approach, employing a gradient-based solution technique, effectively secures global optimal solutions, a characteristic that contributes to aiding the main network in learning a superior feature embedding model. Given this consideration, this study employed the MetaOptNet model as the auxiliary network. Through Meta Learning, it is possible to train a model using a support set that is suitable for a query set, with the learning objective being to train a model capable of adapting to a variety of classification tasks. If the support set samples for each task are derived from the same task’s data, and the query set comes from data with different characteristics, then the learning objective of Meta Learning shifts to learning to classify data from the same task but with different characteristics. We anticipate that the model trained in this manner will demonstrate enhanced generalization capabilities. This training method is designated as Single-Task Meta Learning, which this research utilizes to train the auxiliary network.

### 3.3. Training

During the training phase, each batch consists of 12 color pathology images of size 224 × 224 pixels. The data fed into the auxiliary network are not the original images but the embeddings obtained after feature extraction by the main network, with each embedding having a dimensionality of 2048. Thus, each input image generates one embedding, resulting in a total of 12 embeddings per batch. Half of these embeddings are treated as support samples for the auxiliary network, while the remaining half serve as query samples. 

**Figure 3:**
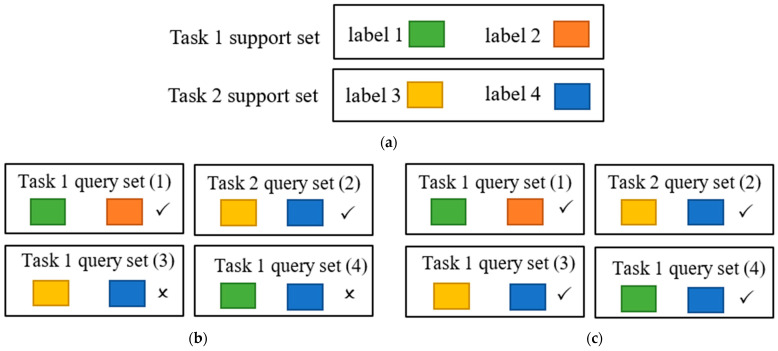
Illustrating the differences between the support sets and their corresponding query sets in Single-Task Meta Learning and Meta Learning: (**a**) support sets for two tasks, (**b**) effective query sets for Meta Learning, where a checkmark indicates a valid combination and a cross indicates an invalid combination, and (**c**) effective query sets for Single-Task Meta Learning.

In terms of model training, the approach of Single-Task Meta Learning is similar to traditional Meta Learning, necessitating the division of the training set into a support set and a query set, with the classification error of the query set quantifying model loss for updating the model parameters. For Single-Task Meta Learning, each support set originates from the same task, allowing for arbitrary combinations of support and query sets. Considering two tasks, designated as Task 1 and Task 2, where Task 1’s support set is labeled as label 1 and label 2, and Task 2’s support set as label 3 and label 4 (as shown in Figure 3a, in which different color blocks correspond to data with different labels). In traditional Meta Learning, during the training of Task 1, the support set can only contain labels 1 and 2, meaning Task 1’s query set can also only correspond to data labeled as 1 and 2 from Task 1, as depicted in Figure 3b. However, in Single-Task Meta Learning, Task 1’s query set could correspond to either data labeled as 1 and 2 from Task 1 or data labeled as 3 and 4 from Task 2, as illustrated in Figure 3c.

The total loss of the overall network $L_{total}$ is defined in Equation (1), in which $L_{aux}$ means auxiliary loss coming from the auxiliary network and $L_{m}$ denotes main loss coming from the main network. Within the provided training dataset $D^{train}={\left\{\left(x_{t},y_{t}\right)\right\}}_{t=1}^{N}$, it can be further divided into support set $D^{support}$ and query set $D^{query}$. The configuration of the support set and the query set aligns with the intentions of Single-Task Meta Learning, ensuring that the image characteristics of these two sets exhibit significant differences. The primary objective of the base learner O-SVM is to deduce the parameters θ for the predictor $y=f\left(x;\theta \right)$, aiming for effective generalization to the unseen query dataset. The domain undergoes a transformation into a feature space facilitated by a feature embedding model $f_{\phi_{e}}$, which is parameterized by $\phi_{e}$, as shown in Figure 1. For learners that rely on optimization, the parameters *θ* are derived by minimizing the empirical loss across the training dataset. This concept is encapsulated in Equation (2). The objective of Single-Task Meta Learning is to learn a feature embedding model $f_{\phi_{e}}$ that minimizes generalization error across breast pathology images with different characteristics given a base learner O-SVM. Formally, the learning objective is expressed in Equation (3). We use the negative log-likelihood of the query data to measure the performance of the feature embedding model $f_{\phi_{e}}$. The meta learning objective of Equation (3) can be re-expressed as Equation (4), where $\omega_{k}$ is the output weight of O-SVM ${(D}^{train};\phi_{e})$ for class *k* and γ is a learnable scale parameter. Regarding $L_{m}$, the cross-entropy loss, which is most commonly utilized in classification tasks, reflects the classification quality of the main network, and it will not be repeated on here. Overall, thanks to the feedback provided by the auxiliary network through loss $L_{aux}$, the feature embedding model ${\;f}_{\phi_{e}\;}$ has the opportunity for further optimization. Meanwhile, the Single-Task Meta Learning strategy enhances the generalization ability of the feature embedding model.

(1) $$L_{total}=L_{aux}+L_{m}$$

(2) $$\theta \;=O-SVM{(D}^{train};\phi_{e})\;={min}_{\theta }L_{aux}\left(D^{train};\theta ,\phi_{e}\right)$$

(3) $${min}_{\phi }\left[L_{aux}\left(D^{query};\theta ,\phi \right)\right]$$

(4) $$L_{aux}\left(D^{query};\theta ,\phi ,\gamma \right)=\sum_{\left(x,y\right)\in D^{query}}\left[-\gamma \omega_{y}\cdot f_{\phi_{e}}\left(x\right)+log\sum_{k}\exp(\gamma \omega_{k}\cdot f_{\phi_{e}}\left(x\right))\right]$$

## 4. Experiments 

**Figure 4:**
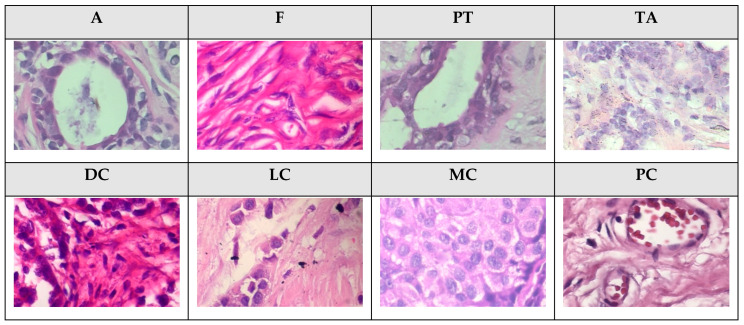
The 400× sample images of eight subcategories of breast tumors.

In this study, the accuracy (ACC) and Dice coefficient of the malignant category were used as efficacy evaluation indicators for the benign/malignant binary classification task. For the subcategories’ classification task, ACC, F1 Score, MCC (Mathews Correlation Coefficient), Kappa, and G-Mean were used as efficacy evaluation indicators. The classification dataset is the BreaKHis dataset [4], which uses H&E staining, the image size is 700 × 400, and the image magnifications are 40×, 100×, 200× and 400×. Each image has a benign/malignant label and the corresponding subcategory label for a total of 7909 images. Pathology images at different magnifications exhibit distinct image characteristics. Utilizing a dataset composed of mixed-magnification pathology images can effectively challenge the generalization ability of the classification models. This study partitions the dataset into 70% for the training set and 30% for the test set, with 25% of the training set being utilized as the validation set. The subcategories of benign tumors are A, Fs, PTs, and TAs. The malignant tumor subcategories are DC, LC, MC, and PC. Figure 4 shows 400× sample images for each subcategory.

### 4.1. Classification Performance

**Figure 5:**
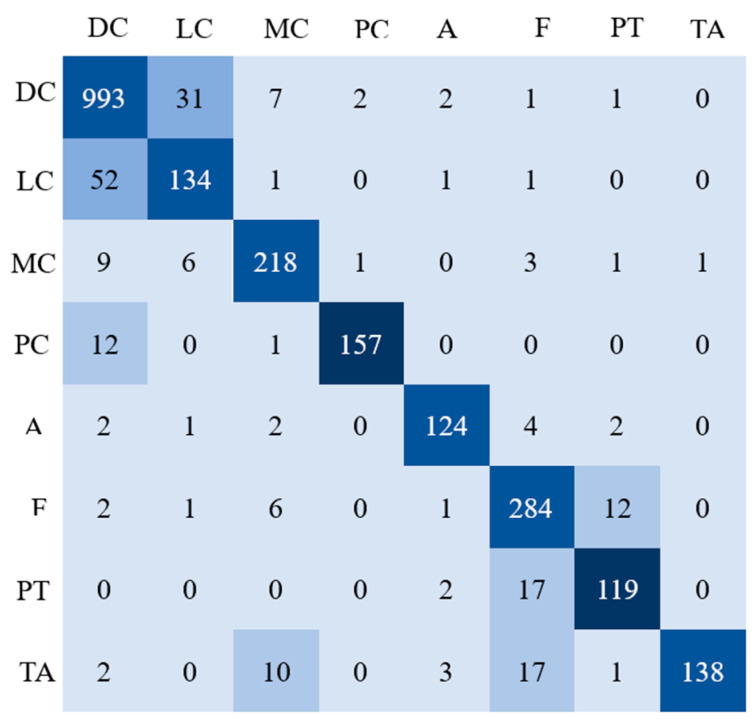
The multi-class confusion matrix of the proposed method.

Table 1 presents the ACC and Dice coefficients of binary classification for benign and malignant breast pathology images utilizing various existing CNN-based methodologies. The results demonstrate that the model introduced in this investigation exhibits superior classification accuracy and Dice coefficient in comparison to the existing CNN-based methods. Additionally, the efficacy of various approaches in discerning between benign and malignant subcategories of breast pathology images is detailed in Table 2. Here too, the findings illustrate that the proposed model surpasses the existing CNN-based methods in terms of subcategory classification accuracy, F1 Score, MCC, Kappa, and G-Mean. This suggests that employing an O-SVM as the auxiliary network, combined with the Single-Task Meta Learning strategy, effectively enhances the model’s generalization capabilities. The confusion matrix for the subcategory classification results of the proposed model is depicted in Figure 5. It reveals that the malignant DC and LC classes are most prone to misclassification, followed by the benign F and PT classes, which also tend to be confused. Additionally, it can be observed that the benign TA class, besides being easily misclassified as the benign F class, may also be mistakenly identified as the malignant MC class.

### 4.2. Ablation Study

To further explore how the selection of different network models as auxiliary and main networks affects the overall system’s classification performance, we designed the following experiment. Given that previous results indicate subcategory classification as a more challenging task, which better reflects the strengths and weaknesses of the models, we proceeded to evaluate models through ablation studies on the subcategory classification task. The main network models selected for comparison include ResNet, SE-ResNet, DenseNet, and ResNeXt, while the auxiliary networks chosen are O-SVM, LPN [38], and Graph Neural Network (GNN) [39], all employing the Single-Task Meta Learning strategy proposed in this study. The results, as shown in Table 3, reveal that ResNeXt as the main network model achieves the best performance, and O-SVM stands out as the most effective auxiliary network. This superiority of O-SVM may be attributed to its optimization through an analytical approach, making it superior to other types of auxiliary networks. Conversely, employing DenseNet as an auxiliary network resulted in poorer performance, possibly due to DenseNet’s architecture, which stacks all preceding network layers, thus amplifying the influence of the initial layers. This direct impact on the feature extraction of the early layers by the auxiliary network can lead to an over-assistance situation. O-SVM, less affected by these issues, can minimize generalization error, thus more readily finding superior solutions.

**Figure 6:**
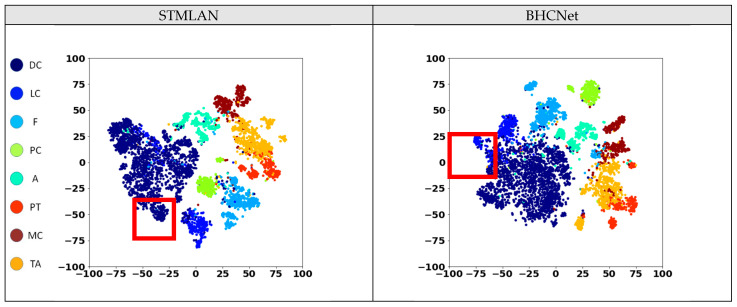
Visualization of the learned features by different models, in which different colors correspond to different categories of tumors. The area highlighted by the red box shows that STMLAN can more clearly separate the feature distribution of different categories such as DC and LC.

To understand the impact of the auxiliary network on feature distribution, we employed t-SNE [40] for the visualization of the learned features by the models. The feature embeddings acquired by both BHCNet [12] and the proposed STMLAN model were compared, with the results being presented in Figure 6, in which different colors correspond to different categories of tumors. The areas highlighted by red boxes show that STMLAN enables a more distinct separation between the feature distributions of different categories, whereas BHCNet exhibits a more pronounced blending of categories. Furthermore, to objectively quantify the degree of separation in feature distribution across different categories, this study employed the Silhouette Score as a metric for assessing the quality of the feature distribution. The data on Silhouette Scores are presented in Table 4. The results indicate that STMLAN achieves the highest Silhouette Score with an increase of 31.85%. It was also observed that the absence of the auxiliary network support in the STMLAN model leads to a reduction in the Silhouette Score to levels comparable with BHCNet. This indirectly demonstrates the effectiveness of the auxiliary network and the Single-Task Meta Learning strategy in optimizing feature embeddings.

**Table 1:** Binary classification accuracy comparison.

Model	ACC (%)	Dice/*p*-Value
CNN [41]	96.00	94.11/0.00
BHCNet [12]	97.36	95.31/0.00
ResNet [42]	93.40	90.13/0.00
NucDeep [43]	96.21	93.21/0.00
ResHist [44]	90.83	88.01/0.00
myResNet-34 [45]	91.67	89.72/0.00
STMLAN	**98.32**	**96.35**/0.00

The bold value indicates the best result.

**Table 2:** Multi-class classification accuracy comparison.

Model	ACC (%)	F1	MCC	Kappa	G-Mean
CNN [41]	80.30	0.82	0.77	0.79	0.76
BHCNet [12]	88.81	0.91	0.86	0.86	0.84
ResNet [42]	78.84	0.80	0.76	0.77	0.75
NucDeep [43]	68.30	0.70	0.66	0.68	0.65
STMLAN	**90.66**	**0.93**	**0.88**	**0.90**	**0.87**

The bold value indicates the best result.

**Table 3:** Multi-class classification performance comparison for auxiliary and main networks using different models (M-Net: main network; Aux-Net: auxiliary network).

	M-Net	ResNet	SE-ResNet	DenseNet	ResNeXt
Aux-Net					
x		87.84	87.35	88.75	89.48
O-SVM [26]		**89.18**	**88.34**	**89.27**	**90.66**
LPN [25]		88.98	87.81	89.25	90.32
GNN [32]		88.94	88.10	88.46	89.92

The bold value indicates the best result.

**Table 4:** Silhouette Scores comparison.

Model	Silhouette Score
BHCNet [35]	0.135
STMLAN w/o auxiliary network	0.145
STMLAN	**0.178**

The bold value indicates the best result.

## 5. Conclusions

In the endeavor to classify breast tumor pathology images, the intrinsic variability present in the characteristics of each pathology image presents a significant challenge for the clinical deployment of CAD systems for breast tumor pathology classification. To address this challenge and develop a system with robust generalization capabilities, this paper introduces the Single-Task Meta Learning Auxiliary Network (STMLAN) model. The STMLAN comprises a main network and an auxiliary network, showcasing exceptional classification capabilities. A pre-trained ResNeXt was utilized as the main network for its superior classification abilities, while MetaOptNet, known for its analytical optimization capabilities, serves as the auxiliary network. Drawing upon the generalization strengths of Meta Learning, the Single-Task Meta Learning strategy was proposed to train the auxiliary network. This approach harnesses the additional optimization momentum provided by the auxiliary network, enabling the training of a feature embedding model within STMLAN that exhibits enhanced generalization capabilities for classification tasks. Through a series of experiments, STMLAN has been proven to outperform other CNN-based classification methods in distinguishing between benign and malignant tumors, as well as classifying eight subcategories. Analysis from the ablation study also reveals that the overall model performance is optimized when employing ResNeXt as the main network in conjunction with O-SVM as the auxiliary network. Furthermore, feature data visualization and Silhouette Scores have both confirmed that training the auxiliary network with the Single-Task Meta Learning strategy indeed allows the main network’s feature embedding model to learn more discriminative and generalizable high-level features from breast tumor pathology images.

## Data Availability

The data presented in this study are available in this article.
